# Predicting Future Organ Support Needs Using Longitudinal Emergency Department Data: A Proof-of-Concept Study

**DOI:** 10.21203/rs.3.rs-9036340/v1

**Published:** 2026-04-03

**Authors:** Samuel Chiacchia, Katie Lebold, Andrew Moore, Hayley Hedlin, Christian Rose, David Kim, Jenny Wilson

**Affiliations:** Stanford Medicine; Oregon Health & Science University; Stanford Medicine; Stanford Medicine; Stanford Medicine; Stanford Medicine; Stanford Medicine

**Keywords:** emergency critical care, organ support, time-series analysis

## Abstract

**Background:**

Prediction of organ support needs, rather than mortality or critical care transfer alone, may improve the utility of early warning scores (EWS). Existing EWS may have limited sensitivity in predicting organ support due to reliance on cross-sectional snapshots of patient physiology, limiting their ability to account for changes in patient status. We aimed to develop and compare novel models capable of using longitudinal clinical data to predict organ support or death (OSD) within 48 hours of hospital admission.

**Methods:**

We leveraged a retrospective cohort of adult ED encounters at a U.S. quaternary academic medical center from March 1, 2022, to February 5, 2024. Encounters were included if patients were ≥ 18 years and admitted to a medical service; those receiving organ support in the ED were excluded. The primary outcome was a composite of vasopressor initiation, invasive mechanical ventilation, continuous renal replacement therapy, or death within 48 hours of admission. Performance metrics included AUROC, AUPRC, sensitivity, and specificity.

**Results:**

1.7% (549/32,329) experienced organ support or death within 48 hours of admission. The transformer-based neural net demonstrated the strongest overall performance, with an AUROC of 0.84 and AUPRC of 0.20, outperforming the baseline to National Early Warning Score 2 (NEWS2) with higher sensitivity for the primary outcome (0.78 vs. 0.61) while maintaining sufficient specificity (0.71 vs. 0.83). XGBoost and elastic-net regression showed similar improvements in sensitivity (both 0.83) with modest reductions in specificity relative to NEWS2 calculated at time of admission.

**Conclusions:**

Organ support represents a potentially modifiable and temporally proximal marker of critical illness. Models trained to interpret longitudinal trends in clinical variables—rather than cross-sectional snapshots—may better mirror clinician reasoning.

## BACKGROUND

The volume and complexity of patients presenting to emergency departments (EDs) have increased substantially, without proportional growth in inpatient capacity.^1^ As a result, emergency physicians face increasing pressure to triage and disposition patients accurately and efficiently. Among admitted patients, unplanned ICU transfers are associated with higher morbidity and mortality, making early identification of patients at risk for near-term decompensation essential for the equitable and effective use of critical care resources^2–8^. Although emergency clinicians excel at recognizing and stabilizing overt critical illness, it remains difficult to determine which patients who appear stable at the time of admission will ultimately require ICU-level care early in their hospital course.

The Society of Critical Care Medicine has emphasized the lack of reliable, objective criteria for ICU triage, while endorsing the use of early warning scores (EWS) to support risk stratification.^9^ Traditionally, EWS development has focused on mortality and ICU transfer as endpoints. Yet mortality endpoints may reflect an unpreventable outcome while failing to identify patients more likely to respond to timely interventions.^10^ Transfer-based endpoints, while more sensitive than mortality, are at risk for bias shaped by institution-specific ICU admission practices and policies, limiting generalizability. Thus, scores like NEWS2 may miss patients who require escalation of care but lack immediate ICU transfer criteria.^10,11^

Shifting focus to prediction of future organ support needs could overcome these limitations and provide important resolution with respect to delivery of specific critical care resources and appropriate disposition at the time of admission.^10^ However, existing early warning scores, like NEWS2, appear limited in their ability to predict near-term organ support needs using clinical data collected from ED patients at the time of admission.^11,12^ We hypothesize that addressing these limitations using novel models that incorporate longitudinal vital sign trends, in addition to laboratory and demographic data, could improve prediction of organ support needs at the time of hospital admission, thereby supporting triage of admitted patients at-risk for near-term decompensation. While similar approaches have been applied in ICU patient populations, few have evaluated such an approach could be applied to ED patient populations^13–17^.

There are multiple model architectures able to process longitudinal and multi-modal data streams; each with their own strengths and limitations. For example, linear regression is easily interpretable but fails to capture non-linear relationships between input data and outcomes of interest and requires feature engineering to first be applied to time-series data. Tree-based models, such as XG-Boost, can capture such non-linear relationships and handle missing data natively, but like regression, require feature engineering to provide temporal context to time-series data. Newer machine learning approaches, such as transformer-based neural nets, are well suited for processing missing and time-series data and can capture non-linear relationships between variables, but require vast amounts of training data and therefore may be limited in prediction of rare clinical outcomes such as organ support or mortality events. To test these strengths and limitations, and to compare performance across different model architectures, we developed three binary classifiers that leverage time series data to predict organ support or death (OSD) within 48-hours of admission from the ED.

## METHODS

### Study Design, Setting, and Population

We conducted a retrospective cohort study using all adult ED encounters at a U.S. quaternary academic medical center between March 1, 2022 and February 5, 2024, where the disposition was admission to an inpatient medical service. Patients were included if they were ≥18 years old and admitted from the ED to a predefined set of adult medical services, including general medicine, subspecialty medicine (e.g., cardiology, hepatology, oncology), and intensive care units. We excluded encounters of patients admitted to surgical or psychiatric services, ED observation units, those with missing patient identifiers, or without an ED evaluation (i.e. direct admissions). Patients admitted to surgical services were excluded due to the different system of care used for the triage and initial management of traumatically injured patients. Patients who received organ support (invasive mechanical ventilation, vasopressors, or continuous renal replacement therapy) or died in the emergency department were excluded from the analysis, as they had already met the endpoint of interest. We treated each ED encounter as unique in our models such that multiple admissions from an individual patient were included and handled as described below. This study was conducted in accordance with the ethical principles outlined in the **Declaration of Helsinki** and was approved by the **Stanford University Institutional Review Board** IRB 69934. The requirement for informed consent was waived due to the retrospective nature of the study and use of de-identified electronic health record data.

### Primary and Secondary Outcomes

The primary composite outcome was organ support or death within 48 hours of hospital admission. Organ support was defined as initiation of vasopressors, invasive mechanical ventilation, or continuous renal replacement therapy (CRRT). Non-invasive positive pressure ventilation and high-flow nasal oxygen were not considered organ support for the purpose of this analysis. Extracorporeal membrane oxygenation (ECMO) was also not included as organ support, as it was assumed patients received other organ support therapies before initiation of ECMO. Secondary outcomes include each type of organ support individually or death within 48 hours of admission.

### Data Collection, Processing, and Partitioning

Structured electronic health record (EHR) data were extracted from the research data warehouse, including demographics, vital signs, laboratory values, and hospital course details relevant to our primary and secondary outcomes including administration of vasoactive medications, mechanical ventilation, continuous renal replacement therapy, or death. Use of vasopressors was identified via continuous infusion orders for norepinephrine, epinephrine, vasopressin, dobutamine, dopamine, or phenylephrine; push-dose pressors were excluded. Receipt of mechanical ventilation was determined using respiratory care flowsheets. Continuous renal replacement therapy was identified by dialysis nursing orders documenting initiation of CRRT. Time-to-event variables were calculated relative to the admission timestamp, and not a patient’s physical location, to assess outcome timing.

Predictor variables were chosen based on previous work exploring performance of longitudinal vital signs and laboratory values in predicting clinical outcomes among ICU patients^18–23^. Charlson comorbidity scores were derived using validated mappings of ICD-9 and ICD-10 codes^24^. To include time-series data in linear and tree based models, minimum, maximum, and composite metrics for ED-based vital signs were calculated. Composite metrics were calculated by calculating the slope of the line of best fit of each vital sign with respect to time multiplied by the R^2 value to control for strength of fit.^25^ Shock index was calculated each time a systolic blood pressure and heart rate were recorded within 5 minutes of each other.

Multiply imputed data were used for the elastic net regression models. We performed predictive mean matching (PMM) multiple imputation using the {mice} package 3.19.0.^26^ Twenty imputations were performed using five iterations per chain. All continuous variables were standardized using z-score normalization prior to imputation.

A 75/15/10 split, constrained to maintain patient-level temporal ordering, for training, validation, and testing sets was used. To prevent data leakage from repeat patient encounters, we implemented a patient-level temporal data split. The dataset was partitioned by medical record number using a temporal ordering approach, where each patient was sorted by earliest ED arrival time, producing. All encounters for a given patient were assigned to a single partition. Training set class distributions could not be balanced due to positive class rarity; thus class imbalance was addressed using a weighted loss function. The validation and test sets retained their natural class distributions for realistic performance assessment. All data collection, processing, partitioning, and subsequent analysis was completed using R 4.5.1.^27^

### Model Development

We trained logistic elastic net regression models to predict the primary outcome within 48-hours after admission using {glmnet} 4.1–10.^28^ Each model was trained using 5-fold time-series cross-validation, with hyperparameters (penalty λ and mixture α) tuned over a predefined grid (α 0–1 by 0.05; λ 10^−4^-10^−1^ by 10^−1/3^). The final model was selected based on the highest area under the precision recall curve (AUPRC) evaluated on validation data.

A gradient-boosted decision tree model was developed using the {xgboost} 3.1.3.1 engine to predict primary outcome within 48-hours.^29^ A Latin hypercube sampling strategy was used to explore combinations of hyperparameters, including number of trees, learning rate, maximum tree depth, and regularization parameters. Model tuning used the same time-series cross-validation strategy as the elastic net regression. The final model was selected based on the highest area under the precision recall curve (AUPRC) evaluated on validation data.

We developed a transformer-based neural network model using the {torch} 0.16.3 to predict the primary outcome.^30,31^ The model processes sequences of up to 150 timesteps containing vital signs, laboratory values, patient demographics, ED length of stay, and Charlson comorbidity index. To preserve information about data availability, we implemented a dual-feature architecture where each clinical variable is accompanied by a binary indicator denoting whether the value was observed (1) or missing (0). Missing values were set to zero after normalization, allowing the model to learn appropriate uncertainty based on data completeness.

Continuous features were normalized using z-score standardization, with means and standard deviations calculated exclusively from observed (non-missing, non-padded) values in the training set; these parameters were then applied to validation and test sets for normalization. The transformer architecture consisted of 4 encoder layers with 2 attention heads, a feedforward dimension of 128, sinusoidal positional encodings to capture temporal ordering, and a 3-layer classification head with ReLU activations and dropout (0.2, 0.1) for regularization. We employed padding masks to exclude completely empty timesteps from attention computations. The model was trained using the AdamW optimizer (learning rate=1×10^−4^, weight decay=1×10^−3^) with binary cross-entropy loss weighted by class frequency to address outcome imbalance. Training proceeded for 20 epochs with early stopping based on validation set precision-recall area under the curve (AUPRC), and gradient clipping (max norm=1) was applied to prevent instability.

### Platt Scaling and Model Performance Evaluation

Model calibration was performed using Platt scaling, where raw logits from the validation set were used to fit a logistic regression model.^32^ Calibrated models were then used to transform raw predictions on the held-out test set into final probabilities. Performance was assessed on the held-out test set using area under the receiver operating curve (AUROC), AUPRC, sensitivity, specificity, and predictive values. The optimal threshold for binary classification was determined using Youden’s J statistic. Model discrimination for secondary outcomes was evaluated using PRC and ROC curves with AUC calculations.

We employed SHapley Additive exPlanations (SHAP) to quantify feature importance and interpretability of model predictions in python.^33^ We generated summary plots displaying the distribution of SHAP values for each feature, where the magnitude indicates importance and the sign indicates the direction of effect on prediction. For continuous features, color-coding represents feature values, revealing non-linear relationships and interaction effects between feature values and their impact on predictions.

### NEWS2 Calculation

NEWS2 was calculated using the closest documented vital signs and blood gas values (arterial or venous) to inpatient admission, defined as the time of entry of an admission order to a medical inpatient service. Hypercapnic respiratory failure was defined as pCO_2_ >45 mmHg (ABG) or >50 mmHg (VBG), in keeping with NEWS2 precedent. Patients without blood gas data were presumed not to be hypercapnic. Per original recommendations, NEWS2 ≥5 or any individual component score of 3 was considered high risk.

## RESULTS

### Cohort characteristics and outcomes

**Figure 1:**
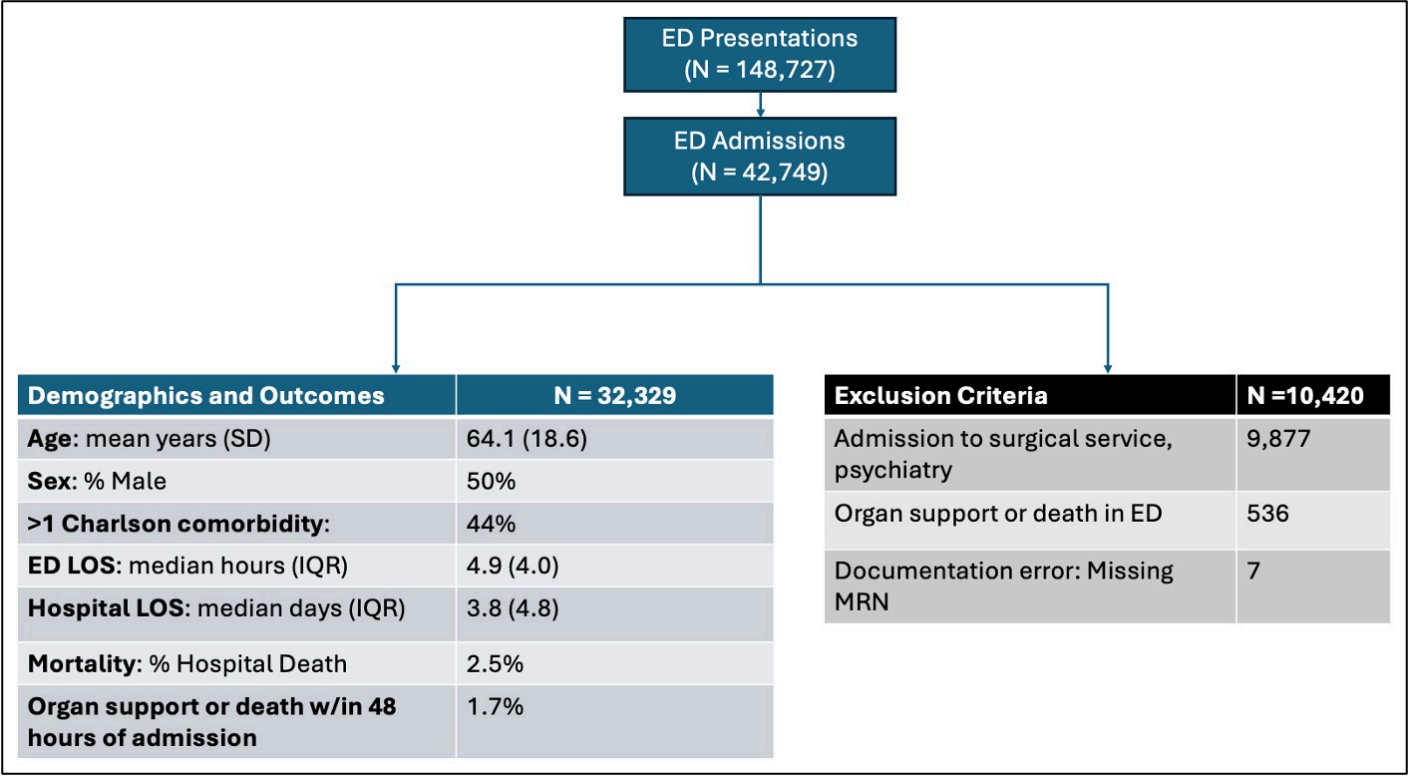
Consort diagram illustrating cohort selection with associated demographics and clinical outcomes.

Of 148,727 adult ED encounters between March 2022 and February 2024, 32,329 (21%) met all inclusion criteria and were used for model development (Figure 1). ED encounters were most commonly excluded due to discharge home (n= 105,978, 71%) or admission to a surgical or psychiatric service (n= 9,877, 6%). Among included patient encounters, the median age was 64 years, 50% were male, and 44% had greater than one co-morbidity included in the Charlson co-morbidity index. Median ED length of stay was 4.9 hours, and median hospital length of stay was 3.8 days. Cohort selection, demographics, and hospital outcomes are further summarized in Figure 1.

**Figure 2:**
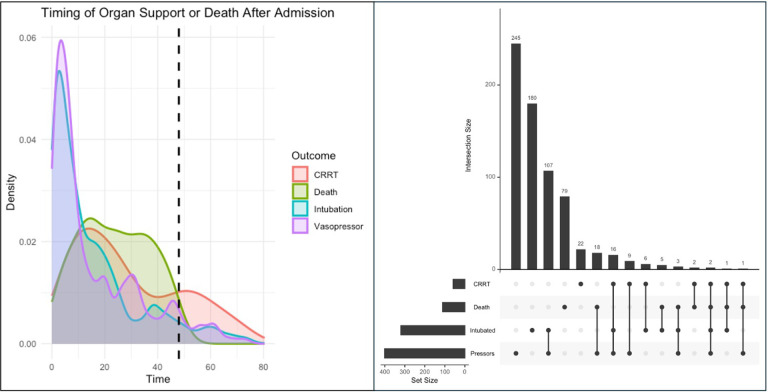
Timing and co-occurrence of critical care outcomes (i.e., vasopressors, intubation, CRRT, or death) among admitted patients. A) Density plot representing distribution of individual critical care outcomes with respect to timing of hospital admission order placement among all patients that experienced organ support or death after admission; x-axis represents time after admission order is placed; y-axis represents probability density of individual critical care outcomes; dashed vertical line indicates 48-hours after admission. B) Upset plot illustrating overlap between critical care outcomes organized by individual critical care outcomex.

Six hundred ninety-six encounters required organ support or resulted in death during their hospital admission, with 576 (81%) meeting the primary outcome of organ support or death within 48 hours of hospital admission (Figure 2A). Patients who received organ support within 48 hours of admission most frequently required only one support modality (Figure 2B). Among subtypes of organ support, vasopressor use (n = 333, 1% of total cohort) and invasive mechanical ventilation (n = 304, 0.9%) were most common, followed by CRRT (n = 45, 0.1%). There were only 34 deaths within 48 hours of admission (0.1%).

Patients who required organ support or died within 48 hours of admission had similar age to those that did not meet our primary outcome (66 years [43–89] vs 66 years [39–93]) but were more likely to be male (56% vs 50%) and have at least one chronic co-morbidity (56% vs 43%). ED length of stay was shorter among encounters with decompensation (median 3.9 hours [3.4] vs 5.0 hours [4.0]) while hospital length of stay was longer (8.0 days [11.9] vs 3.8 days [4.8]).

### Performance and feature importance across model architectures is similar

The 32,329 samples in our cohort were randomly assigned to training (n = 26,346, 81%), validation (n = 3796, 12%), and test (2,187, 7%) sets. The prevalence of primary and secondary outcomes in training, validation, and test sets were similar to those observed in the full cohort (Supplemental Figure 1).

We trained logistic elastic net regression models to predict the primary outcome within 48-hours after admission. Hyperparameter tuning over a predefined grid (α 0–1 by 0.05; λ 10^−4^-10^−1^ by 10^−1/3^) identified the highest AUPRC of 0.12 for ENR with alpha 0.3 and lambda 0.04. AUPRC for validation and test sets was 0.07 (Supplement Table 1). For gradient-boosted decision tree models, a Latin hypercube sampling strategy was used to explore combinations of hyperparameters, including number of trees, learning rate, maximum tree depth, and regularization parameters. The highest AUPRC of 0.15 was achieved with a model with 652 trees of depth 3, learn rate of 5.81×10^−7^, and loss reduction 3.3×10^−6^. AUPRC for validation and test sets were 0.08 and 0.12, respectively (Supplement Table 1). The small learning rate reflects convergence of the tuning procedure to the lower bound of a wide log-scaled search range. When the learning rate was constrained to conventional XGBoost values (0.001–0.1), model discrimination was largely unchanged (AUPRC 0.19), suggesting performance was not sensitive to this parameter. Finally, we trained a transformer using the AdamW optimizer (learning rate=1×10^−4^, weight decay=1×10^−3^) with binary cross-entropy loss weighted by class frequency to address outcome imbalance. Training proceeded for 20 epochs; the strongest performing model had an AUPRC of 0.35. AUPRC for validation and test sets were 0.14 and 0.2 respectively (Supplement Table 1).

Insight into feature importance was guided by model architecture. For ENR, we used the absolute value of the model coefficients to assess feature importance; for XGB, we used average gain (i.e. the mean improvement in model loss attributed to splits on each feature across all trees); for the transformer we used SHapley Additive exPlanations (SHAP). Similar features were important to model performance across all three architectures, specifically trends in systolic blood pressure, heart rate, respirations, pulse oximetry, as well as lab values such as venous pH, lactate, and blood urea nitrogen (Supplemental Figure 2).

**Figure 3:**
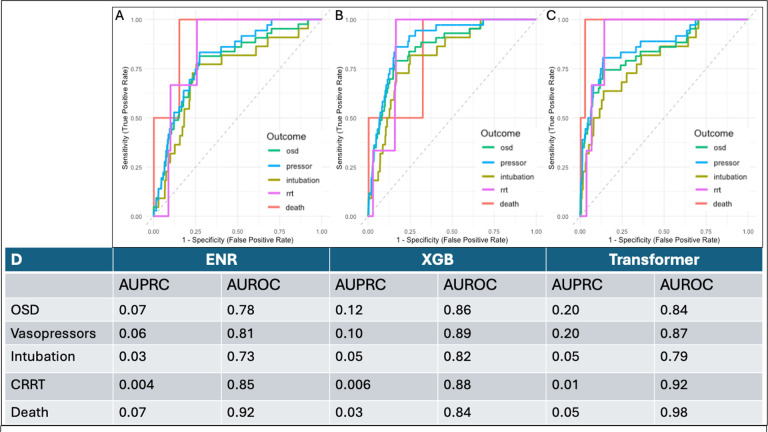
Comparison of Model Performance by AUROC and AUPRC. A,B,C) ROCs for hold-out test sample (N = 2,187) generated by ENR (A), XGB (B), and Transformer (C) models in prediction of organ support or death (OSD) or individual critical care outcomes within 48 hours of admission. D) Table summarizing AUPRC, AUROC by model architecture and outcome.

Figure 3 summarizes model performance in predicting our primary and secondary outcomes. In the prediction of our primary outcome among samples in the hold-out test set, the transformer had the highest AUPRC (0.2) while the XGB had the highest AUROC (0.86). Among secondary outcomes, performance was strongest in prediction of future vasopressors and CRRT and weakest in prediction of invasive mechanical ventilation and mortality across all model architectures (Figure 3).

### Times series models are more sensitive than NEWS2 in predicting organ support or death within 48 hours of admission

**Figure 4:**
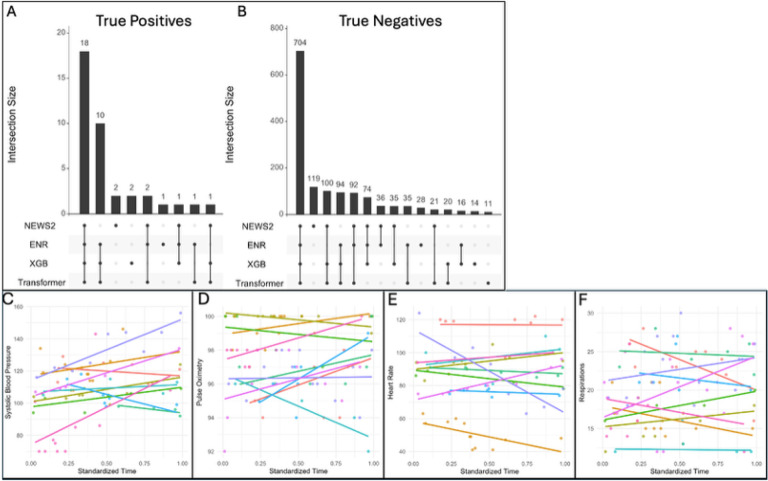
NEWS2 at Time of Admission vs Time Series Models in Prediction of Organ Support or Death A-B) Upset plot illustrating discordance in true positives and true negatives in our hold out test set. C-F) Vital signs used for NEWS2 calculation with respect to relative collection time during ED course among 10 patients correctly identified by all three time series models but missed by NEWS2 at time of admission; sample intersect denoted in Figure 4A by red asterix.

Previously, we evaluated the performance of NEWS2 in prediction of future organ support or death after admission.^12^ To compare performance of NEWS2 at time of admission to our time series models, we subset our test sample (n = 2,187) to include samples in which a NEWS2 score could be reliably calculated using our previous methods.^12^ Among this subset of 1,545 patient encounters, 43 (2%) met our primary outcome of organ support or death within 48 hours of admission (Figure 4A).

Of the 43 cases that met our primary outcome, 14 (33%) cases correctly identified by at least one time series model were missed by NEWS2 calculated at time of admission; 2 (4%) cases correctly flagged by NEWS2 were missed by all three time series models. Of the 1,497 encounters that did not involve organ support or result in death within 48 hours, 218 (14%) correctly identified by a time series model were missed by NEWS2 calculated at time of admission; 119 (7%) encounters correctly flagged by NEWS2 were missed by all three time series models.

All three time series models had greater sensitivity than NEWS2 in prediction of our primary outcome as well as use of vasopressors or invasive mechanical ventilation within 48 hours of admission (78–83% vs 61%, Table 1). NEWS2 had greater specificity in prediction of our primary outcome (78% vs. 70–71%). Assessment of performance in prediction of CRRT and death in this sub-group was limited by the low prevalence of CRRT and death.

### Case series analysis of discordant classifications illustrates limitations of cross-sectional risk scores like NEWS2

While feature importance highlights the role of longitudinal trends in driving model performance for predicting near-term organ support (Supplemental Figure 2), we sought to directly visualize how temporal changes in vital signs may result in misclassification of high risk patients by cross-sectional risk scores, like NEWS. To accomplish this, we conducted a case series analysis of positive samples with discordant predictions between NEWS2 and our time-series models. Figure 4A–4B illustrate discordant true positive and true negative predictions between NEWS2 and the time series models. We examined the 10 samples correctly predicted by all three time-series models to meet OSD within 48 hours of admission that were not identified by NEWS2 calculated at the time of admission (Figure 4A). We plotted the vital signs used to calculate NEWS2 with respect to time (Figure 4C–F). These figures illustrate vital sign trends over time; these trends are not captured by cross sectional risk scores and could explain differences in sensitivity observed in Table 1.

## DISCUSSION

In this retrospective study of approximately 32,000 ED admissions, we explored whether real-world, longitudinal clinical data improved prediction of organ support or death within 48-hours of admission relative to the existing cross sectional risk score, NEWS2. A central contribution of this work is the use of organ support, rather than mortality or ICU transfer alone, as a primary endpoint. Organ support represents a potentially modifiable and temporally proximal marker of critical illness. Unlike mortality, which may reflect irreversible disease processes, or ICU transfer, which is influenced by local policy and bed availability, initiation of vasopressors, mechanical ventilation, or CRRT more directly reflects physiologic decompensation requiring intervention. Aligning prediction models with such actionable endpoints may improve relevance for future interventional studies.

Second, we hypothesized that longitudinal modeling better aligns with clinician reasoning, and observed similar discrimination and improved sensitivity relative to a cross-sectional score (NEWS2), suggesting that incorporating physiologic trajectories may provide incremental predictive information beyond admission-time snapshots. The similar discrimination achieved across regression, tree-based, and transformer models suggests that the predictive signal may lie primarily in the longitudinal physiologic information itself rather than in any specific modeling architecture. This finding is important, as it indicates that improvements in prediction may stem from better alignment of features with clinical trajectories rather than increasing algorithmic complexity.

While encouraging, discrimination in the “80/80” range implies substantial misclassification. At low outcome prevalence (1.7%), even modest false-positive rates generate a large number of alerts relative to true events, raising questions about acceptable tradeoffs between sensitivity and alert burden. Our study was not designed to determine whether this level of misclassification is clinically acceptable, nor whether the model adds value beyond careful physician assessment. Future work must quantify the net clinical benefit across plausible risk thresholds and compare model performance directly against clinician gestalt in prospective settings.

This study has several strengths. First, we analyzed a contemporary patient cohort from a large quaternary care center, capturing real-world clinical practice and preserving outcome prevalence in validation and test sets. Second, the use of patient-level temporal splits minimized data leakage and provide a rigorous assessment of generalizability. Third, we present a novel framework for predicting organ support outcomes by demonstrating models trained to predict composite outcomes (i.e. vasopressor, intubation, CRRT, or death) are also able to predict individual organ support outcomes with comparable sensitivity and specificity. This has important implications for future efforts to predict organ support that may be constrained by limited outcome prevalence, proving especially relevant for deep learning models like transformers. Fourth, we compared three distinct modeling paradigms against an existing risk score, NEWS2. Others have shown similar superior performance of machine learning models in predicting ICU admission, however, we chose organ support or death, which is subject to less institutional and provider variability and to increase generalizability.^34–36^ Finally, this analysis aimed to compare performance across different model architectures, a crucial step prior to external validation and deployment.

This study also has important limitations. It was conducted at a single academic medical center, limiting evaluation of generalizability to settings with different patient populations, resource availability, and ED workflows. The two-year timeframe constrained the number of positive cases and class imbalance may have influenced model behavior despite use of weighted loss functions. Although we selected a composite endpoint to address concerns that critically ill patients often require multiple organ support modalities, many patients in our cohort required only one form of support within the 48-hour window. Finally, although transformers demonstrated encouraging discrimination, their performance likely remains sensitive to the quality, density, and ordering of time-series data. Missingness patterns in EHR data are non-random and may encode clinical decision-making rather than pure physiology; while our dual-feature representation attempts to capture this, it may not fully resolve sampling bias. Finally, our models were trained on structured EHR data drawn from a heterogeneous population encompassing diverse disease states and organ failures. While this reflects real-world practice, it limits mechanistic insight and biological interpretability. Future efforts integrating physiologic trajectories with molecular or disease-specific data may offer greater explanatory power and potentially improved discrimination.

## CONCLUSIONS

Organ support represents a potentially modifiable and temporally proximal marker of critical illness. Models trained to interpret longitudinal trends in clinical variables—rather than cross-sectional snapshots—may better mirror clinician reasoning. Our findings support the hypothesis that incorporating longitudinal data improves sensitivity in predicting near-term organ support or death.

## Supplementary Material

Supplementary Files

This is a list of supplementary files associated with this preprint. Click to download.

- SUPPLEMENTAL.docx

## Figures and Tables

**Table 1:** Comparison of Test Characteristics Among Times Series Models and NEWS2

	NEWS2		ENR		XGB		Transformer	
Test Sample (N = 1,545)	Sensitivity	Specificity	Sensitivity	Specificity	Sensitivity	Specificity	Sensitivity	Specificity
OSD (n = 38)	0.61	0.78	0.83	0.71	0.83	0.70	0.78	0.71
Vasopressors (n = 33)	0.60	0.78	0.85	0.71	0.90	0.70	0.85	0.71
Intubation (n = 21)	0.57	0.77	0.76	0.70	0.76	0.69	0.67	0.70
CRRT (n = 3)	0.67	0.77	1	0.70	1	0.69	1	0.70
Death (n = 2)	1	0.77	1	0.70	0.5	0.68	1	0.70

## Data Availability

The datasets generated and analyzed during the current study are not publicly available due institutional data privacy policies but are available from the corresponding author on reasonable request.
